# Care Me Too, a Mobile App for Engaging Chinese Immigrant Caregivers in Self-Care: Qualitative Usability Study

**DOI:** 10.2196/20325

**Published:** 2020-12-02

**Authors:** Mandong Liu, Tongge Jiang, Kexin Yu, Shinyi Wu, Maryalice Jordan-Marsh, Iris Chi

**Affiliations:** 1 Suzanne Dworak-Peck School of Social Work University of Southern California Los Angeles, CA United States; 2 Edward R Roybal Institute on Aging University of Southern California Los Angeles, CA United States; 3 Leonard Davis School of Gerontology University of Southern California Los Angeles, CA United States; 4 Epstein Department of Industrial and Systems Engineering Viterbi School of Engineering University of Southern California Los Angeles, CA United States

**Keywords:** mHealth, co-design, usability, acceptability, immigrant, caregiver, mobile phone

## Abstract

**Background:**

Caregiving and self-care are challenging for Chinese immigrants in the United States due to limited accessible support and resources. Few interventions exist to assist Chinese immigrant caregivers in better performing self-care. To address this gap in the literature, our team developed the Care Me Too app to engage Chinese immigrant caregivers in self-care and conducted a user experience test to assess its usability and acceptability.

**Objective:**

This paper aims to report the results of the app’s usability and acceptability testing with Chinese immigrant caregivers and to solicit participants’ feedback of the app design and functions.

**Methods:**

A total of 22 Mandarin-speaking Chinese caregivers participated in the study, which consisted of 2 parts: the in-lab testing and the 1-week at-home testing. In-depth face-to-face interviews and follow-up phone interviews were used to assess user experience of the app’s usability and acceptability and to solicit feedback for app design and functions. Directed content analysis was used to analyze the qualitative data.

**Results:**

Among the 22 participants, the average age was 60.5 (SD 8.1) years, ranging from 46 to 80 years; 17 (77%) participants were women and 14 (64%) had an associate degree or higher. Participants reported uniformly positive ratings of the usability and acceptability of the app and provided detailed suggestions for app improvement. We generated guidelines for mobile health (mHealth) app designs targeting immigrant caregivers, including weighing flexibility versus majority preferences, increasing text sizes, using colors effectively, providing engaging and playful visual designs and functions, simplifying navigation, simplifying the log-in process, improving access to and the content on the help document, designing functions to cater to the population’s context, and ensuring offline access.

**Conclusions:**

The main contribution of this study is the improved understanding of Chinese caregivers’ user experiences with a language-appropriate mHealth app for a population that lacks accessible caregiving and self-care resources and support. It is recommended that future researchers and app designers consider the proposed guidelines when developing mHealth apps for their population to enhance user experience and harness mHealth’s value.

## Introduction

### Asian American and Pacific Islander Caregivers’ Needs

Caring for others can be rewarding but also straining. The negative impact of caregiving is well documented in the literature, and it contributes to higher levels of stress and depression and lower levels of physical health and self-efficacy compared with noncaregivers [1-3]. Caregiving is especially challenging for Asian American and Pacific Islander populations. Asian American and Pacific Islander populations are the second fastest growing ethnic group among the aging population, with the number of older adults projected to increase by 145% from 2010 to 2030 [4]. However, stereotyped as the “model minority,” Asian American and Pacific Islander individuals’ caregiving and service needs are often overlooked [5]. According to a meta-analysis study in 2005, Asian American and Pacific Islander caregivers performed a higher number of caregiving tasks, were more depressed, had worse physical health, and used less formal support than White caregivers [6].

Language barriers, employment situations, and cultural beliefs heighten Asian American and Pacific Islander caregivers’ challenges. Asian American and Pacific Islander caregivers reported a lack of language-appropriate and culturally sensitive formal services [7-9]. Almost 1 in 5 Asian American and Pacific Islander caregivers preferred non-English to English materials [10]. Asian American and Pacific Islander family caregivers were more likely to work (67%) and work full-time (61%) compared with other ethnic groups [10]. It is common in Asian American and Pacific Islander cultures to not speak up about challenges faced or ask for external caregiving help due to a strong sense of pride in self-managing [8,11].

The Chinese population comprises the largest Asian group in the United States. [12]. Literature shows that Chinese immigrant caregivers face severe caregiving challenges [9,13]. Providing culturally and linguistically appropriate caregiving and self-care support is crucial for enhancing the well-being of Chinese immigrant caregivers, but resources specific to their needs are lacking. In an evidence-mapping study, only 7 training programs were found to be provided to Chinese immigrant caregivers; not all the programs included self-care topics [14].

To address this research gap, our team, consisting of experts from the fields of social work, nursing, and gerontology, developed an in-person Chinese caregiver self-care training program and pilot tested it in Los Angeles county. The program was based on the body-mind-spirit (BMS) model to address a caregiver’s holistic well-being [15]. The BMS model finds its roots in the Eastern philosophies of Taoism (eg, yin-yang perspectives) and Buddhism and focuses on the dynamics between individuals and the world and on mind-body and human-nature harmony. It also adopts relevant knowledge from traditional Chinese medicine. The model regards the physical, emotional, and spiritual as indivisible yet distinctly different aspects of the same reality. With these concepts being familiar to Chinese populations, the BMS model is believed to cater to the unique needs of Chinese immigrants, with cultural adaptability and consistency [15]. Researchers have used the BMS model in developing interventions and obtained preliminary evidence that sheds light on the feasibility of using the BMS model to tackle the stress and vulnerability inherent in the process of caring [16].

During the pilot testing of the in-person training, the team encountered challenges in recruitment and scheduling because of caregivers’ time constraints and transportation barriers, making the training difficult to access for our target audience. This calls for training delivery that allows flexibility in access options to respond to the special needs of Chinese immigrants.

### Mobile Health Apps for Caregivers

An increasing number of web-based interventions and mobile health (mHealth) apps have been developed to deliver more accessible resources for older adults and caregivers [17]. According to 2 systematic review studies (one with 7 studies and the other with 14 studies), web-based interventions and mHealth apps were effective in decreasing caregivers’ depression and anxiety levels and increasing self-efficacy [18,19]. A study found that 7 out of 10 caregivers were somewhat or very receptive to using smartphone apps to assist them in caregiving [20]. However, in 2017, only 44 out of over 200,000 mHealth apps were designed especially for caregivers, and only 10 included self-care support functions, such as app-based support groups, a stress tracker, and health maintenance advice [17]. To the best of our knowledge, there is no app designed for Chinese immigrant caregivers of limited English proficiency to help them better care for their physical and psychological well-being.

**Figure 1:**
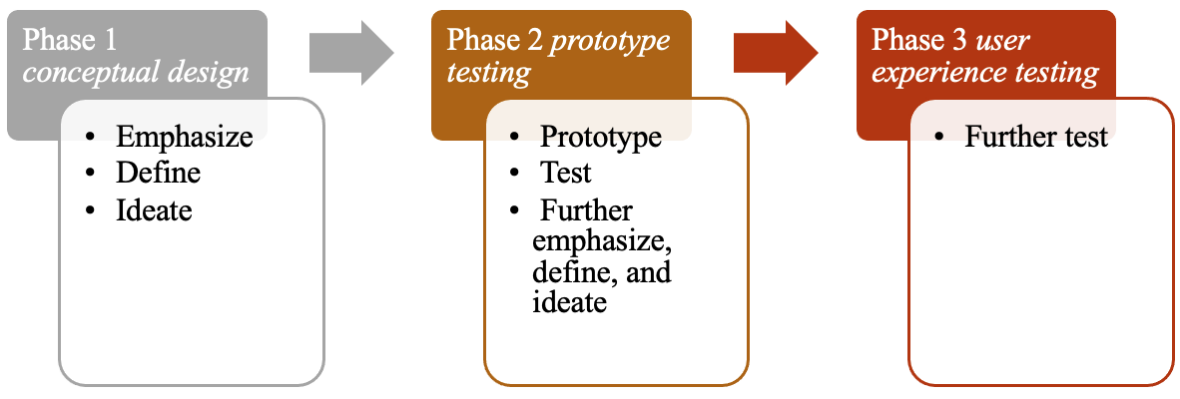
The 3 phases of participant co-design based on the design thinking process.

Using a co-design approach in collaboration with local Chinese immigrant caregivers, our team of researchers, consisting of experts from the fields of social work, nursing, gerontology, engineering, and information technology, developed the Care Me Too app in Chinese for promoting caregivers’ self-care. The team followed a traditional 5-stage design thinking process in co-designing the app: emphasize (develop understanding of the users’ needs), define (frame the issue in a human-centric manner), ideate (develop a breadth of ideas), prototype (produce a sample or prototype), and test (test the components of the overall app design and study the feasibility of the app as a solution for caregivers’ self-care) [21,22]. We conducted the design thinking process in 3 phases of participant co-design. In phase 1, the conceptual design phase, we interviewed 7 Chinese immigrant caregivers to emphasize, define, and ideate user needs. The research team then designed a Care Me Too app prototype. In the phase 2, the prototype testing phase, we interviewed the same 7 participants plus 5 additional Chinese immigrant caregivers to review the prototype design and gauge feasibility to help caregiver self-care. The interviews helped the research team further emphasize, define, and ideate user needs to refine the app design. The research team then developed the functional Care Me Too app. In phase 3, the user experience testing phase, the team assessed the usability, acceptability, and at-home use of the Care Me Too app and its specific functions. Figure 1 illustrates these 3 phases. This paper aims to report the results of the app’s user experience testing with Chinese immigrant caregivers, in which existing literature is limited, and to solicit participants’ feedback for app design and functions.

When designing the Care Me Too app, the team took into consideration Nielsen’s 10 usability heuristics [23] to enhance the interaction design. Those heuristics most relevant to our app included match between the system and the real world (speak the users’ language and use words, phrases, and concepts familiar to them), user control and freedom (a clearly marked “emergency exit” to leave the unwanted state or to undo), consistency and standards (use the same words and actions for the same thing to decrease confusion), aesthetic and minimalist design (decrease irrelevant information), and help and documentation (easy-to-search help and documentation should be provided).

## Methods

### Care Me Too App Description

In the current literature, the most widely adopted functions in web-based interventions for caregivers included information and resources, assistance in problem solving, peer psychosocial support, professional psychosocial support, and family communication and care coordination [17,19]. Based on the research team’s health promotion and digital expertise, we designed the app with 6 functions: required readings, BMS exercise videos, extended readings, a chat room, coaching, and a community resource inventory. The content of the required readings, which focused on self-care knowledge and skills, was modified after evaluating our BMS in-person caregiver training program and was divided into 4 chapters: (1) the role and stress of caregivers (eg, different caregiving duties, boundary issues, sources of stress, and an overview of using the BMS model to achieve holistic health); (2) diet, exercise, sleep, and medication management (eg, how to achieve a balanced diet, how to correctly store and use medication); (3) problem-solving skills (eg, how to increase self-enhancing thinking, a 7-step guide to problem solving with 2 real-life examples); and (4) how-to information on using formal and informal support resources (eg, what formal and informal resources are, things to pay attention to when immigrants use these resources). Interactive graphs were also used to better illustrate the information. For example, a healthy eating plate was used to visually assist caregivers in achieving a balanced diet [24]. After a user clicks a type of food on the plate (eg, fruits), a textbox pops up and describes its nutrition and recommended serving per day.

The BMS videos were visual demonstrations of exercises aiming to promote exercise and help relieve caregiving stress, such as head and neck self-massage, breathing exercises, and guided imagery. Extended readings addressed caregiving knowledge and skills, such as communication, oral health, legal issues, and more.

Social features have been employed in mHealth apps and have been shown to be effective in helping users gain social support and decrease stress related to their illnesses [25-27]. Therefore, we developed 2 functions, a chat room and coaching, to promote communication among caregivers and between caregivers and health coaches (health care professionals or researchers). To reduce learning burden in the testing stage, instead of building a new in-app chat room, we chose to use WeChat, a commonly used messaging and social media app among Chinese populations. For the coaching function, caregivers could schedule a meeting to consult a coach about health or caregiving-related questions; however, no real coaching took place during the testing.

**Figure 2:**
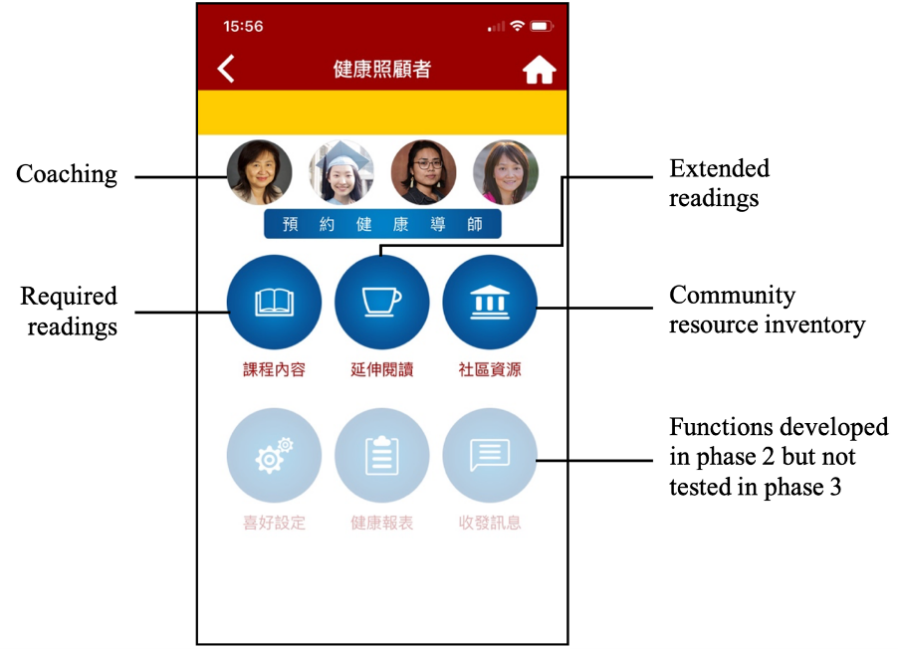
Home screen of the Care Me Too app.

The community resource inventory contained a variety of local and national resources available to Chinese caregivers and older adults, such as the Chinese American Coalition for Compassionate Care. Each organization was presented with a brief introduction, their website link, and their contact information. Figure 2 shows the app’s home screen.

### Participants and Recruitment

Caregivers were recruited in the Los Angeles area from January to April 2019. The inclusion criteria were (1) 18 years or older, (2) ability to speak and understand Mandarin Chinese, (3) experience caring for someone aged 65 or older for at least 3 months, (4) experience assisting the care recipient with at least one activity of daily living or a medical task, and (5) possession of an iPhone (the team could provide one if a caregiver did not have an iPhone). A participant could either be a formal caregiver (eg, home care aide) or an informal caregiver (eg, family member, friend).

The team sent out flyers to community-based organizations with Chinese immigrant clients (eg, senior housing, community service centers, adult day health care centers) and asked their staff members to help recruit participants. Contact information for those interested in participating was directed to the project coordinator for eligibility screening. The coordinator then scheduled an interview time with caregivers who were eligible. Potential participants were also encouraged to refer their friends to participate. A total of 22 caregivers were recruited and participated in the testing.

### User Experience Testing

The testing consisted of 2 parts: the 1- to 2-hour in-lab testing and the 1-week at-home testing. In the in-lab testing, which was observed by team members, participants could verbalize their feelings and thoughts while using the app prototype. The at-home testing provided participants with an opportunity to fully explore the app prototype and read the health literacy content.

Each in-lab testing was conducted in Mandarin Chinese either in a private room in a university or a private office at a local senior housing site by 2 Chinese-speaking graduate student research assistants, one serving as the instructor and interviewer and the other as the notetaker. There was a total of 5 research assistants involved, all trained by senior researchers in the team on qualitive interviews. They strictly followed the predesigned task lists and interview guides (described below) to ensure fidelity. The instructor explained the project once again before the participant signed the informed consent and reported their sociodemographic information. After that, the instructor helped download the app to the participant’s cell phone and introduced app functions based on a predesigned task list (eg, read a portion of the content, turn pages, view in-text graphics). This 12-item task list was developed by team members with an engineering background based on the app prototype functions. A hard copy help document in Chinese was presented to the participants for their reference. After hearing instructions, the participant went through the same task list on their own to show mastery of each task. During this process, the instructor provided assistance only if needed. For example, if a participant forgot how to go back to the home screen, the instructor reminded the participant of the icon that looked like a house. The participant needed to master all tasks before moving on to the next step. In the end, the interviewer interviewed the participant using a predesigned interview guide (see Multimedia Appendix 1). Specifically, a 10-item app usability scale modified after the System Usability Scale [28] was used to measure usability; open-ended interview questions were also used to elicit participants’ opinions on the app prototype design and functions, such as the usefulness and ease of use of each app function, the visual appeal, the advantages and disadvantages of using an app to receive training, and the barriers to and facilitators of using the app. Sample questions were “Do you feel it was easy or difficult to use the following functions? Why?” and “What will help you use this app more conveniently?” Participants also provided detailed feedback on the health literacy reading content and its cultural sensitivity, with results reported in a future paper. The whole process was audio- and videorecorded. The notetaker took notes during the whole process. Upon completion of the in-lab testing, each participant was given a US $50 gift card for a commonly used grocery store among Chinese immigrants.

After the in-lab testing, the participant went home with a different 8-item app use task list (eg, complete one assigned chapter of the required readings, watch exercise videos) to be completed in the next 7 days. After a week, either the instructor or the notetaker who conducted the in-lab testing with the participant called the participant to complete a 30-minute phone interview for further evaluation of the app. Closed- and open-ended questions were used regarding the participant’s actual use of the app, ratings of and more detailed suggestions for each app function, and the overall app’s helpfulness, usefulness, and visual appeal (see Multimedia Appendix 2). Each participant was given a US $100 grocery store gift card upon completion of the 1-week at-home testing and the phone interview. After finishing all interviews, the research team summarized findings in a table and forwarded the table to participants for confirmation and further feedback, if any.

### Analysis

All 22 in-lab testing interviews were transcribed verbatim in Chinese. The team adopted the directed content analysis approach proposed by Hsieh and Shannon [29] for the analysis of the qualitative data. Two investigators (ML and TJ), who were graduate students and had been trained by senior researchers on qualitative analysis, developed the initial coding scheme based on existing app functions and Nielsen’s 10 usability heuristics [23]. After that, they began to independently code transcripts using the predetermined codes. As new information emerged from the transcripts, the coders either modified their current coding scheme or created new codes. The 2 coders discussed their coding results and identified disagreements twice a week. Any unresolvable discrepancies were discussed at the weekly meetings with the research team until a final decision was achieved. All 22 transcripts were separately and independently coded by 2 coders, with a κ coefficient of 0.92. NVivo 12.1.0 software (QSR International) was used for analysis. After deciding the final findings and making results tables, ML translated the findings into English; findings tables in English were then reviewed by the whole research team, including the investigator MJM, a native English speaker. Questions regarding English terms and phrases used were resolved in team meetings.

All activities were approved by the university’s institutional review board.

## Results

### Participant Characteristics

The participants’ average age was 60.5 (SD 8.1) years, ranging from 46 to 80 years. Of the 22 participants, 17 (77%) were women and 14 (64%) had an associate degree or higher. A total of 16 of the 22 (73%) participants were formal nonfamily caregivers and 6 (27%) were family caregivers. On average, the participants had lived in the United States for 20.4 (SD 10.2) years, ranging from 5 to 40 years. In addition, 14 of the 22 (64%) participants spoke little or no English. All had experiences using an iPhone even though 2 were using an Android phone during the interview. Of the 22 caregivers, 11 (50%) used an iPad or a tablet and 10 (45%) used a computer or a laptop in daily life.

### Feedback for Specific App Functions During In-Lab Testing

Participants’ positive feedback and suggestions for specific app functions during the in-lab testing are summarized in Table 1 (quotes are available upon request). Participants reported that the current 5 functions could be beneficial to them in gaining knowledge on caregiving, self-care, exercising, enhancing their communication, connection, and obtaining support from other caregivers and the coaches. They provided detailed improvement suggestions for each function, such as making the content audible; being able to highlight, copy, save, and share the content; being reminded to exercise; categorizing coaches based on their sociodemographic background or expertise; and categorizing community resources based on the type of service. They also suggested alternative format preferences. For example, to communicate with coaches, instead of using video chat (as originally considered), some preferred talking on the phone and some preferred texting. Participants hoped to add a certificate function that would allow them to be awarded a certificate whenever they completed a skill training (eg, blood pressure measuring) to show to a potential employer. One participant suggested adding in-app games that care recipients and caregivers could play together to gain reward points. See Table 1 for a detailed list of comments.

**Table 1:** Feedback for app functions.

Function	Positive feedback	Suggestions
Required and extended readings	1. Participants could learn how to better care for themselves and care recipients. - 1.1. Participants gained a better understanding of holistic health: body, mind, and spirit.	1. Be able to highlight, copy, and save content in the app and share it with others. 2. Be able to print the content from one’s phone. 3. Have a place in the app to show all the latest content updates. 4. Use less text. 5. Some participants preferred listening to over viewing the text; some suggested using videos to illustrate the content; some suggested having multiple formats (text, audio, and video) from which participants could choose. - 5.1. If audio is used, it should be in a storytelling style.
BMS^a^ videos	1. Short BMS videos would assist caregivers in exercising conveniently. 2. The video content was credible. 3. Watching videos was more fun than reading the text.	1. Encourage caregivers and care recipients to exercise together. 2. Some participants wanted to be reminded to exercise. However, reminders should be fun and entertaining.
Coaching	1. Participants could ask questions and get expert advice. 2. Participants could gain connection and emotional support.	1. Provide a clear function description, such as instructions on when to contact coaches and how much it costs. 2. Add an introduction for each coach. - 2.1. Categorize coaches based on sociodemographic information. 3. Some participants preferred talking on the phone; some preferred texting. If using video chat, participants preferred WeChat over FaceTime. Finally, some mentioned that participants should be allowed to choose to use different platforms (texting, phone call, video chat) for different needs.
Chat room (WeChat as platform)	1. WeChat group could enhance caregivers’ communication on caregiving and health-related issues. 2. It was a platform for asking app-related questions and providing feedback to the research team.	1. Provide a clear function description, such as the purpose of having a caregiver WeChat group. 2. Monitor the content of the discussion. 3. Change name tags in the chat group to indicate whether the user is a research team member or caregiver. 4. Some did not want to talk to strangers and thus might not use the chat room function; some would only talk when asking questions; some preferred one-on-one communication to group chat.
Community resource inventory	The function could assist caregivers in seeking help and gaining support.	1. Provide Chinese resources. 2. Categorize resources. 3. Add emergency information and show it on the front page of this function.
Certificate (brought up by participants)	A certificate would be proof of skill training.	1. Divide the training into several sessions. 2. Provide a certificate for each skill that is learned. 3. Set a high bar for obtaining a certificate.
Games and rewards (brought up by participants)	N/A^b^	1. In-app entertaining games could be designed for care recipients and caregivers to play and spend time together. 2. Points could be gained through game playing and used to exchange for rewards.

^a^BMS: body-mind-soul.

^b^N/A: not applicable.

### Feedback and Reflection on Overall App Design During In-Lab Testing

Participants’ feedback for the overall app design during the in-lab testing is summarized in Table 2 under the categories of visibility, navigation and error prevention, password and privacy, language and terms, and help and documentation (participants’ direct quotes are available upon request). Overall, the majority of participants reported that it was easy to navigate the app and use the current help document. Many liked the current color theme (ie, cardinal, gold, and blue). Participants provided detailed feedback to improve the app design, such as enlarging the font size in different ways, using lighter colors, having a clear log-out icon, and using terms and phrases that are less professional. Again, participants expressed alternative design preferences. For example, there were suggestions regarding the format of the help documentation. In-app text, in-app instruction videos, and a PDF file on the computer were mentioned to replace the current hard copy help document. For the password, some preferred to have a password while others stated that a password was not necessary.

**Table 2:** Feedback for overall app design.

Themes	Design heuristics	Positive feedback	Suggestions
Visibility	1. Aesthetic and minimalist design 2. Match between system and the real world	Some participants liked the current color theme.	1. Font: - 1.1. Use a bigger font size. - 1.2. Have a function to change the font size. - 1.3. Be able to view horizontally - 1.4. Develop an iPad version of the app. - 1.5. Connect the phone to a television. 2. Color: - 2.1. Use lighter colors. - 2.2. Use more green. - 2.3. Use comforting colors. - 2.4. Increase contrast. 3. Add the university’s logo. 4. Make the appearance more interesting, such as adding pictures.
Navigation and error prevention	1. Flexibility and efficiency of use 2. Match between system and the real world 3. Error prevention 4. Help users recognize, diagnose, and recover from errors.	1. It was easy to navigate the app. - 1.1. Easy to use the home button, go forward and backward, and scroll up and down 2. Navigation was familiar from other apps.	1. Have a table of contents to go directly to a specific page. 2. Have a clear log-out icon. 3. Use pop-ups for additional information in the readings. 4. Use the scrolling bar instead of a drop-down list to select date and time for the meeting. 5. For the “reselect” icon in answers for case studies: - 5.1. Darken its color. - 5.2. Make it more visible. 6. For clickable text in graphics: - 6.1. Have a clear “close page” icon. - 6.2. Use arrows or other ways to highlight what to click.
Password and privacy	Recognition rather than recall	The password requirement was valued by some participants.	1. Password should be automatically saved. 2. Participants should be able to change their password. 3. Regarding the password format, some liked to use their phone number as the password (current version) and some preferred to use a fingerprint. 4. Some said a password was not needed. - 4.1. A password would be needed if the app is not free.
Language and terms	Match between systems and real world	Some participants said the terms and expressions used were understandable.	1. Pay attention to different expressions in different regions of China. 2. Use language and terms that are less professional.
Help and documentation	Help and documentation	The current help document (hard copy) was easy to use.	1. Some preferred a hard copy; some preferred the in-app text version; some wanted an in-person orientation; some preferred an instruction video; some wanted to have a PDF document on the computer. 2. Some participants wanted a more comprehensive help document, while some said a help document was not needed.

Participants reported that three main advantages of using a mobile app for receiving training were convenience, having thorough educational content in one place, and enhancing communication with others through content sharing. The disadvantage was the lack of personal contact with other participants. Their perceived biggest barrier to using the app was a caregiver’s busy work and personal life; other barriers included having no Wi-Fi or cellular data plan and the potential cost of use in the future. Perceived facilitators to app use to be enhanced in future versions included (1) an app introduction that clearly explains the app’s purposes and potential benefits to caregivers, (2) enhancing the app’s relevance to care recipients, (3) delivering different training content based on caregivers’ backgrounds, (4) maintaining a reasonable reading load or letting caregivers learn at their own pace, and (5) providing a sense of accomplishment, which could be fulfilled by the certificate function.

### Usability Scale Results

As assessed by the 10 items of the usability scale, participants were generally positive about the app’s usability, with the mean score for each item ranging from 3.86 to 4.32 (score range of 1-5, with higher scores being better). The 3 items that scored below 4 were “Most people would learn to use this app very quickly,” “I need a technical person’s support to be able to use the app,” and “There was too much inconsistency in this app” (reversed score).

### Participants’ App Use at Home and Follow-up Assessment

The 1-week at-home testing and follow-up assessment were completed by 21 of the 22 participants; 1 participant did not complete the testing due to her busy schedule. On average, participants reported that they used the app 5.6 days in 1 week (range of 1-7, median of 7) and spent 26.8 minutes on it per day (range of 10-90, median of 20). All but 1 participant rated the app “good” or “very good.” A total of 76% (16/21) of participants thought the app’s color was appealing. Almost half (10/21), however, thought the font size was too small. In addition, 90% (19/21) of participants were interested in using this app in the future after it is fully developed, and all participants were willing to recommend the app to other people. See Table 3 for detailed information.

**Table 3:** App ratings after the 1-week at-home use.

Questions		Values
How many days in the past week did you use the app, mean (SD)		5.6 (2.0)
On average, how many minutes per day did you use the app, mean (SD)		26.8 (19.4)
**Overall, how would you rate our app, n (%)**		
	Very good	11 (52)
	Good	9 (43)
	So-so	1 (5)
**Is the app’s font size appropriate, n (%)**		
	Appropriate	11 (52)
	Too small	10 (48)
**Is the app’s color appropriate, n (%)**		
	Yes	16 (76)
	No	5 (24)
**Did you use the app all by yourself, n (%)**		
	All by myself	20 (95)
	Needed a lot of help from others	1 (5)
**What do you like the most about this app, n (%)**		
	Curriculum	14 (67)
	Functions	2 (10)
	Navigation	1 (5)
	Speed	1 (5)
	Other (convenience, can communicate issues with others, like all above)	3 (14)
**What do you dislike the most about this app, n (%)**		
	Visual appeal and feeling	6 (29)
	Other (prefer to listen; need more updates of the content; would like to have more consultations regarding health, diet, and healthy recipes; exercise videos not easy to find)	6 (29)
	Nothing disliked	9 (43)
**Have you ever used other similar health apps, such as “Health” on an iPhone, n (%)**		
	Yes	4 (19)
	No	17 (81)
**(If answered “yes” to previous question) How is our app compared to other health apps, n (%)**		
	As good as other apps	2 (50)
	Better than other apps	2 (50)
**Are you interested in using this app in the future, n (%)**		
	Interested	19 (91)
	Not sure	1 (5)
	Not interested (reason: already learned the content)	1 (5)
Will you recommend this app to others in the future (yes), n (%)		21 (100)

## Discussion

### Principal Findings

In this study, participants reported uniformly positive ratings of usability and acceptability of the Care Me Too app and its functions. Besides the current functions, participants hoped the team could add a certificate function and a game function in future versions to increase the app’s usefulness and ability to engage. They reported that convenience was the biggest advantage of learning through an app because the format catered to their busy schedules. This is consistent with the literature, in which time restraints have been well documented for Chinese caregivers [30].

Participants provided a range of ideas and suggestions for designing a self-care app for Chinese immigrant caregivers. We summarized their suggestions into general mHealth app development guidelines to enrich the app design literature and benefit other immigrant caregiver populations.

### General: Weigh Flexibility Versus Majority Preferences

Participants had various preferences for the functions and designs of this app. They reported that it would be convenient if we provided different options so that they could choose the most appropriate one depending on their context. For example, one participant stated she would prefer video chatting with the coach if she were not busy; if she were busy, she would text the coach her questions and wait for the reply while continuing with her work. This is consistent with previous research, in which Mortenson and colleagues [26] reported that formal caregivers desired flexibility in the tools within a user self-management app so that the tools could be tailored to users’ individual needs. Thus, researchers need to weigh flexibility versus majority preferences; they can either provide as many options as possible or choose the most widely accepted option, whichever is more appropriate after considering the team’s resources and technical challenges. In the future, researchers and app developers might begin by inventorying resource constraints and weighting them when choosing functions, add-ons, and the degree of tailoring possible.

### Visibility

#### Increase Text Sizes

In the app, we used 14-point font for titles and 12-point font for text, but some participants reported these font sizes to be small. There could be two explanations. First, our participants were middle-aged and older adults. Only 5 of 22 were 50 years or younger, and several reported declining vision. Morey et al [31] reviewed mHealth apps for older adults and suggested that apps for older adults use at least 20-point font [31]. Second, Chinese characters use strokes, which make characters complex and detailed. Also, unlike in English, where there is space between words, no space exists between Chinese characters in one sentence. Thus, text can be cluttered if the font size is too small [32]. A font size larger than 14 points for caregiver mHealth apps in the future might make the app more accessible, considering that many family caregivers are older care recipients’ spouses [10]. Overtly providing functions for choosing the font size or zooming in on the text would be an alternative.

#### Use Colors Effectively

Participants provided different opinions on alternatives to our current app color theme. Some liked the current colors, as they were simple and plain, while some hoped for lighter colors, indicating a desire to allow customization. Participants reported low contrast in some textboxes, which made text difficult to read. For example, for the healthy eating plate, participants reported a lack of contrast between text and some textboxes’ background colors, as well as between the exit icon and the background colors. Morey et al [31] also noted that color contrast for text and background and for icons and background should be high enough that text and icons are easily seen [31]. In short, colors for an app should be selected to enhance user interface design and support usability and engagement.

#### Provide Engaging and Playful Visual Designs and Functions

Another suggestion from participants was to increase user engagement and the app’s playfulness by using more pictures, using kittens and puppies for BMS exercise reminders instead of an alarm, and adding in-app games that caregivers and care recipients could play together to gain points. Adinugroho [33] observed that playfulness was important for improving the “stickiness” of digital products among Asian populations, with icons and emoticons being comical, cute, and cartoony [33]. For game-oriented designs, our finding is consistent with Jessen et al [34], which found that app users welcomed “gameful” designs, such as having one’s own avatars, setting goals to accomplish, and earning rewards, such as points and badges [34]. Meanwhile, gamefully designed tools need to fit users and the context in which they will be used [34]. In our case, participants spend much time with care recipients, and thus, in-app games could be designed for mHealth apps to allow collaboration between caregivers and care recipients so that they learn and have fun together.

### App Navigation

#### Simplify Navigation

Participants confirmed that our app had simple navigation design features that could facilitate universal user uptake. The match between the system for a new app and the real world is clearly significant for users to master the use of a new app and reduce cognitive burden. Our participants reported many of our app designs to be consistent with those in apps that they had used previously, which increased the efficiency of learning to use our app. These features included a home page to allow seeing everything on one screen, a clear home button to quickly get back to the home page if a user gets lost, a scroll bar to move up and down, and arrows to go forward and backward between chapters and sections. Participants also preferred to use a scroll bar, as in the iPhone’s alarm function, instead of using the current drop-down list to select the date and time for coach meetings. Features for us to improve and for others to consider included adding a clear app log-out icon and ensuring crucial information is seen without the need to scroll down. For example, in the current case study textboxes, the reselect icon was sometimes outside of one screen, and participants did not know to scroll down to see the icon without the team members’ assistance. Morey et al [31] also suggested using a back icon instead of a back arrow to decrease confusion, although our participants appeared satisfied with the back arrows in our app.

#### Simplify Log-in Process

Privacy has been a concern in previous mHealth literature for apps in which participants’ information was recorded in the app and shared with health care professionals [35]. However, in this study, privacy did not appear to be an issue, perhaps because our current app does not have a health tracker function and participants did not need to input their personal information (except for their mobile phone number, which was used to log in to the app). Thus, some participants stated there was no need for a log-in password. Some other participants welcomed the use of their phone number as the log-in password. This is similar to smart phone users’ habits in China, where many websites and apps also use mobile phone numbers as the primary identifier for logging in and password recovery [32]. Some participants hoped the app would automatically remember the password if one were required. In brief, a simple and clear log-in process would save users time and make the app use more efficient for caregivers if no privacy concerns exist.

#### Improve Access to and Content of the Help Document

Instead of using a hard copy of the help document, participants proposed that the next version could use a variety of formats, including a hard copy option, in-app text, introduction videos, and a PDF document that they could save on their computer. Some participants also wanted to see a more detailed help document. Considering that future users of our app and other mHealth apps could be novice or inexperienced smartphone users, it is important to provide a step-by-step guide for first-time users. Additional walk-through instructions, such as bubbles for using a new function, could be used, as suggested in previous research [31].

### Function

#### Design Functions to Cater to the Population’s Context

It is crucial to consider locomotion (ie, mobile users use their device while on the move) in the app design to enhance user experiences [36,37]. Our caregivers reported a desire to listen to education materials instead of reading the text so that they could learn while doing household chores. Another context dimension to consider is the immediacy, meaning that mobile users expect to have the right app at the right time [36]. This was manifested in our study when participants reported wanting to add emergency information, such as a 911 link, to the front page of the community resource inventory because of health and medical emergency concerns when caring for older adults. Finally, for mHealth apps, it is important to design app functions with a social circle beyond direct users in mind. In our case, caregiver participants wanted to use the app as a tool to meaningfully spend time with care recipients and suggested adding in-app multiplayer games, as discussed above, as well as changing the title of “BMS exercises” to “BMS exercises that caregivers can do with their care recipients.” They also wanted to have a function to share the educational content with others, such as family members or caregiver colleagues, even though they were not directly related to their caregiving job. This finding is consistent with previous literature on web-based support for Chinese caregivers of dementia patients, in which participants liked to share information with other people in their social network to enhance mutual support [30].

#### Ensure Offline Access

One reported barrier to the use of our app was having no data or Wi-Fi at home or in certain locations. Ensuring offline access would entail that information and resources be downloadable once participants logged in to the app. The BMS videos could not be watched without Wi-Fi or data use because YouTube was the platform used. Chat room and coaching functions could not be used without Wi-Fi or data streaming, either. In the future, there may be legal ways to store YouTube videos to increase accessibility across geographic locations.

### Strengths and Limitations

This study had multiple strengths. One was using a sample consisting of both family and nonfamily caregivers. Previous Chinese caregiver literature tended to focus on the needs of family caregivers only, but nonfamily caregivers are crucial to providing support for older Chinese immigrants, and they experience indistinct boundaries between themselves and their care recipients, with shifts from an occupational relationship to a personalized connection, such that the caregiving relationship becomes similar to a mother-daughter relationship [38]. This creates similarities between Chinese family and nonfamily caregivers. The second was the research team’s expectation of a co-design model, which was shared with participants. The research team had extensive experience in working with older adults and Chinese immigrants.

There were also limitations. The app is currently only available on the iOS platform and in Chinese; due to time restraints of the pilot study, some standard features were not included in the tested version, such as in-app adjustment of the font sizes and a text-to-speech function, which generated much feedback from participants. The assessments were self-reported, and thus, our participants may have reported more positive findings. We did not collect any information on behavior changes activated by app use.

### Conclusions

There is a need to design and develop mHealth apps for caregivers of all populations to help them take care of themselves. This will require attention not only to content but to functionality, ease of use, and pleasure in the use of the app. In this study, using both in-person and at-home testing, we elicited Chinese caregiver participants’ feedback on the functions and design of the Care Me Too app. Caregivers gave positive ratings to the overall experience of using the app. The researcher–expert designers made adjustments in a formative design model and developed guidelines intended to enhance the design, usage, and usability characteristics of subsequent versions and, potentially, of other mHealth apps for immigrants and caregivers. In the future, we will include ways to measure the impact of using the app on caregivers’ health promotion behaviors and changes in their work with care recipients. We will examine ways to incorporate changes proposed by the co-designer immigrant participants to increase engagement, minimize burden, and promote behavior change. Later, we will test the effectiveness of the Care Me Too app in increasing caregivers’ caregiving and self-care knowledge, skills, and self-efficacy and on decreasing negative psycho-socio-spiritual outcomes. We recommend that future researchers and app designers consider the proposed guidelines when developing mHealth apps for their populations to enhance user experience and harness mHealth’s value.

## Appendix

Multimedia Appendix 1 In-lab Testing Interview Guide.

Multimedia Appendix 2 At-Home Testing Interview Guide.

## Abbreviations

BMS: body-mind-spirit
mHealth: mobile health
