# Supplementary material for: Care Me Too, a Mobile App for Engaging Chinese Immigrant Caregivers in Self-Care: Qualitative Usability Study
Source: JMIR Form Res. 2020 Dec 2;4(12):e20325. doi: 10.2196/20325 (PMC7744258; doi:10.2196/20325)
Supplement: Multimedia Appendix 1 [file formative_v4i12e20325_app1.docx]

**In-Lab Testing Interview Guide**

Date________________ Time: ____________________Participant ID ______________

Now I want to ask you some questions to understand your app usage over the past hour.

Part A. Please tell me if you agree with the following statements:

| [Statement](https://cui.unige.ch/isi/icle-wiki/_media/ipm:test-suschapt.pdf) | Strongly disagree | [disagree](https://cui.unige.ch/isi/icle-wiki/_media/ipm:test-suschapt.pdf) | [neutral](https://cui.unige.ch/isi/icle-wiki/_media/ipm:test-suschapt.pdf) | [agree](https://cui.unige.ch/isi/icle-wiki/_media/ipm:test-suschapt.pdf) | [Strongly agree](https://cui.unige.ch/isi/icle-wiki/_media/ipm:test-suschapt.pdf) |
| --- | --- | --- | --- | --- | --- |
| I think I would like to use the app frequently. |  |  |  |  |  |
| I found this app unnecessarily complex. |  |  |  |  |  |
| I found this app easy to use. |  |  |  |  |  |
| I think that I would need the support of a technical person to be able to use the app. |  |  |  |  |  |
| I found the various functions in the app were well integrated. |  |  |  |  |  |
| I thought there was too much inconsistency in this app. |  |  |  |  |  |
| [I would imagine that most people would learn to use this app very quickly.](https://cui.unige.ch/isi/icle-wiki/_media/ipm:test-suschapt.pdf) |  |  |  |  |  |
| I found the app very cumbersome to use. |  |  |  |  |  |
| I felt very confident using the app. |  |  |  |  |  |
| I needed to learn a lot of things before I could get going with this app. |  |  |  |  |  |

Part B

1. Do you feel it was easy or difficult to use the following functions? Do you have other feelings when using them?
   1. log in
   2. view the “help” manual presented in paper format
   3. return to the home page
   4. make an appointment with the coach
   5. view the curriculum content
   6. watch the exercise videos
   7. view the extended reading
   8. view the community resources
   9. comment in the caregiver WeChat group
2. Overall, is it easy or difficult for you to use this app? Please explain in detail.
   1. is it smooth to use the app
   2. is there anything that’s confusing?
   3. is the font size and color appropriate?
3. Do you think this app will benefit you as a caregiver?
4. Does this app lack any functions that you would like to have? What kind of functions?
5. What will help you use this software more conveniently?
   1. prompt only if needed: the “help” manual in the app; families or friends’ assistance in using the app
6. What will make this app more helpful to you?
   1. prompt only if needed: online coach; online caregiver support group
7. Are you interested in using this app in the future? Why or why not？
8. What would prevent you from using this app?
   1. prompt only if needed: log in process; busy daily schedule; not interested in the content; not interested in the exercises; not using WeChat
9. What improvements can we make to the app? e.g., adding other functions, changing the color, fonts, content, etc.
10. What are the advantages and disadvantages of using the app to provide caregiver training, compared to other formats? e.g., taking in-class courses
11. Which group(s) of people do you think this software will benefit?
    1. Prompt only if needed: For example, age group, education level, family or formal caregivers
