# Supplementary material for: Care Me Too, a Mobile App for Engaging Chinese Immigrant Caregivers in Self-Care: Qualitative Usability Study
Source: JMIR Form Res. 2020 Dec 2;4(12):e20325. doi: 10.2196/20325 (PMC7744258; doi:10.2196/20325)
Supplement: Multimedia Appendix 2 [file formative_v4i12e20325_app2.docx]

**At-Home Testing Interview Guide**

Hello. In order to help us improve our Care Me Too program and the app in the future, we’d sincerely appreciate it if you share your valuable comments and suggestions.

Part A. Overall Assessment

Do you agree with the statement below?

|  | Strongly disagree | Disagree | Neutral | Agree | Strongly agree |
| --- | --- | --- | --- | --- | --- |
| A1 The Care Me Too program was helpful to me. | o | o | o | o | o |

A2.1 Have you completed the curriculum content that you were required to read?

o All completed (jump to A2.2)

o Partially completed (jump to A2.1.1)

o Not at all (jump to A2.1.2)

A2.1.1 Why did you only partially complete the required content? e.g., did not have time, did not know how to use, could not see clearly, could not understand the content, not interested, etc.

A2.1.2 Why didn't you read? e.g., did not have time, did not know how to use, could not see clearly, could not understand the content, not interested, etc.

|  | Extremely Difficult | Not easy | So so | Easy | Very easy |
| --- | --- | --- | --- | --- | --- |
| A2.2 Is the content easy to understand? | o | o | o | o | o |

|  | Very unappealing | Unappealing | So so | appealing | Very appealing |
| --- | --- | --- | --- | --- | --- |
| A2.3 Is the content appealing? | o | o | o | o | o |

|  | Not helpful at all | Not helpful | So so | Helpful | Very helpful |
| --- | --- | --- | --- | --- | --- |
| A2.4 Is the content helpful? | o | o | o | o | o |

A2.5 What do you think of the expected length and duration: one week to complete one unit?

o One week is too short

o Just right

o One week is too short

A2.5.1 What suggestions do you have regarding curriculum content? Please give an example:______

A2.6 Have you read the rest of the curriculum content (i.e., not just required chapters)?

o Yes (jump to A3.1)

o Whether

A2.6.1 Why didn't you read? e.g., did not have time, did not know how to use, could not see clearly, could not understand the content, not interested, etc.

A3.1 Have you watched the exercise videos that you were asked to watch?

o Watched all required videos (jump to A3.2)

o Watched some of the videos (jump to A3.1.1)

o Didn't watch the videos at all (jump to A3.1.2)

A3.1.1 Why did you only watch some of the videos? e.g., did not have time, did not know how to use, could not see clearly, could not understand the content, not interested, etc.

A3.1.1 Why didn’t you watch the videos? e.g., did not have time, did not know how to use, could not see clearly, could not understand the content, not interested, etc.

|  | Extremely Difficult | Difficult | So so | Easy | Very easy |
| --- | --- | --- | --- | --- | --- |
| A3.2 Is the content of the exercise videos easy to follow? | o | o | o | o | o |

|  | Very unappealing | Unappealing | So so | appealing | Very appealing |
| --- | --- | --- | --- | --- | --- |
| A3.3 Are exercise videos appealing? | o | o | o | o | o |

|  | Not helpful at all | Not helpful | So so | Helpful | Very helpful |
| --- | --- | --- | --- | --- | --- |
| A3.4 Are exercise videos helpful? | o | o | o | o | o |

A3.5 What suggestions do you have regarding exercise videos? Please give an example._________

A4.1 Have you read the extended reading?

o Yes (jump to A4.2)

o No (jump to A4.1.1)

A4.1.1 Why didn’t you read the extended reading? e.g., did not have time, did not know how to use, could not see clearly, could not understand the content, not interested, etc.

|  | Extremely Difficult | Difficult | So so | Easy | Very easy |
| --- | --- | --- | --- | --- | --- |
| A4.2 Is it easy to understand the content of the extended reading? | o | o | o | o | o |

|  | Very unappealing | Unappealing | So so | appealing | Very appealing |
| --- | --- | --- | --- | --- | --- |
| A4.3 Is the extended reading appealing? | o | o | o | o | o |

|  | Not helpful at all | Not helpful | So so | Helpful | Very helpful |
| --- | --- | --- | --- | --- | --- |
| A4.4 Is the extended reading helpful? | o | o | o | o | o |

A4.5 What suggestions do you have re: extended reading? Please give an example.________

A5.1 Did you try making an appointment with a coach?

o Yes (jump to A5.2)

o No (jump to A5.1.1)

A5.1.1 Why didn’t you make an appointment? e.g., did not have time, did not know how to use, could not see clearly, not interested, not necessary, etc.

|  | Extremely Difficult | Difficult | So so | Easy | Very easy |
| --- | --- | --- | --- | --- | --- |
| A5.2 Is it easy to use the current design to make an appointment with the coach? | o | o | o | o | o |

|  | Very unappealing | Unappealing | So so | appealing | Very appealing |
| --- | --- | --- | --- | --- | --- |
| A5.3 Is this function appealing? | o | o | o | o | o |

A5.4 What suggestions do you have regarding the coaching function? Please give an example:________

A6.1 Did you speak in the caregiver WeChat group?

o Yes (jump to A6.2)

o No (jump to A6.1.1)

A6.1.1 Why didn’t you speak in the weChat Group? e.g. did not have time, did not know how to use, not interested, not necessary, did not like to talk to strangers, etc.

|  | Extremely Difficult | Difficult | So so | Easy | Very easy |
| --- | --- | --- | --- | --- | --- |
| A6.2 Is it easy to use our caregiver WeChat group? | o | o | o | o | o |

|  | Very unappealing | Unappealing | So so | appealing | Very appealing |
| --- | --- | --- | --- | --- | --- |
| A6.3 Is it appealing to use our WeChat group? | o | o | o | o | o |

|  | Not helpful at all | Not helpful | So so | Helpful | Very helpful |
| --- | --- | --- | --- | --- | --- |
| A6.4 Is our WeChat group helpful? | o | o | o | o | o |

A6.5 What suggestions do you have regarding making an appointment with the coach? Please give an example. ________________

Part B. App Evaluation

|  | Very bad | Not good | So so | Good | Very good |
| --- | --- | --- | --- | --- | --- |
| B1.1 Overall, how would you rate our app? | o | o | o | o | o |

|  | It's too small | Just right | It’s too big |
| --- | --- | --- | --- |
| B1.2 Is the app’s font size appropriate? | o | o | o |

B1.3 Is the app’s color appropriate?

o It’s appropriate (jump to B2.1)

o It's not appropriate (jump to B1.3.1)

B1.3.1 How to improve the color design? _____________________________

B2.1 How many days in the past week did you use the app?

B2.2 On average, how many minutes per day did you use the app?

B3.1 Did you use the app all by yourself?

o All by myself (jump to B4)

o Needed other’s help but only a little bit (jump to B3.1.1)

o Needed a lot of help (jump to B3.1.1)

B3.1.1 What functions did you need help with? ______________________________

B4 What do you like best about this app?

o Speed (app speed was high)

o Stability (did not experience errors)

o Navigation (smooth use; able to quickly find the functions you wanted to use)

o Functions (app functions met your needs)

o Appearance and feeling (color, font size, and page turning were satisfying)

o Content (curriculum content, exercise videos, extended readings were satisfying)

o Other reasons, please explain (jump to B4.1)

o I like none of the above

B4.1 Other reasons, please explain_______________________________________________

B5 What do you dislike the most about this app?

o Speed (app speed was low)

o Stability (experienced errors)

o Navigation (not smooth use; unable to quickly find the functions you wanted to use)

o Functions (app functions did not meet your needs)

o Appearance and feeling (color, font size, and page turn were unsatisfying)

o Content (curriculum content, exercise videos, extended readings were unsatisfying)

o Other reasons, please explain (jump to B5.1)

o Nothing disliked

B5.1 Other reasons, please explain_______________________________________________

B6 Have you ever used other similar health apps, such as the "health" app on an iPhone?

o Yes

o No (jump to B7)

B6.1 How is our app compared to other health apps?

o Better than other apps

o As good as other apps

o Not as good as other apps

B7 Are you interested in using this app in the future, after it’s fully developed?

o Interested

o Not sure

o Not interested (jump to B7.1)

B7.1 Why aren’t you interested? e.g., not have time, not practical ______________

B8 Will you recommend this app to others (e.g. family, friends) in the future?

o I will

o Not sure

o I will not

Part C. Do you have any other suggestions for improvement regarding our project and the app?
